# Analysis of RNA Modifications by Second- and Third-Generation Deep Sequencing: 2020 Update

**DOI:** 10.3390/genes12020278

**Published:** 2021-02-16

**Authors:** Yuri Motorin, Virginie Marchand

**Affiliations:** 1 Université de Lorraine, CNRS, IMoPA (UMR7365), F54000 Nancy, France; 2 Université de Lorraine, CNRS, INSERM, IBSLor (UMS2008/US40), Epitranscriptomics and RNA Sequencing Core Facility, F54000 Nancy, France

**Keywords:** RNA modification, epitranscriptome, deep sequencing, massive parallel sequencing, single-molecule sequencing, nanopores, RT signature, antibody, methylation, pseudouridine, 2’-O-methylation

## Abstract

The precise mapping and quantification of the numerous RNA modifications that are present in tRNAs, rRNAs, ncRNAs/miRNAs, and mRNAs remain a major challenge and a top priority of the epitranscriptomics field. After the keystone discoveries of massive m^6^A methylation in mRNAs, dozens of deep sequencing-based methods and protocols were proposed for the analysis of various RNA modifications, allowing us to considerably extend the list of detectable modified residues. Many of the currently used methods rely on the particular reverse transcription signatures left by RNA modifications in cDNA; these signatures may be naturally present or induced by an appropriate enzymatic or chemical treatment. The newest approaches also include labeling at RNA abasic sites that result from the selective removal of RNA modification or the enhanced cleavage of the RNA ribose-phosphate chain (perhaps also protection from cleavage), followed by specific adapter ligation. Classical affinity/immunoprecipitation-based protocols use either antibodies against modified RNA bases or proteins/enzymes, recognizing RNA modifications. In this survey, we review the most recent achievements in this highly dynamic field, including promising attempts to map RNA modifications by the direct single-molecule sequencing of RNA by nanopores.

## 1. Introduction

Post-transcriptional RNA modifications (also called “Epitranscriptomics”) can be detected in RNA while using various methods and approaches exploiting the chemical and physico-chemical properties of these non-canonical RNA nucleotides. In addition to classical RNA techniques, such as 5’/3’ and specific internal labeling as well as nucleoside/RNA oligonucleotide analysis by Liquid Chromatography coupled to Mass Spectrometry (LC-MS) or tandem Mass Spectrometry (LC-MS/MS), methods that are based on second- (abbreviated as NGS for Next Generation Sequencing) and third- (NNGS, for Next-Next Generation Sequencing) generation sequencing become increasingly popular. These approaches aim to provide single-nucleotide resolution for the identification of the modified RNA position, but they may be less accurate in the exact nature of the modified residue due to a rather generic treatment used during the library preparation step. The most popular and reliable methods using NGS analysis rely on various specific chemical treatments that are applied to specifically alter RNA-modified residues to make them detectable either as RT-stop or as a mis-incorporation of nucleotides into cDNA. Actually, NNGS approaches mostly use ion-current profiles through the nanopore or kinetics of deoxynucleotide triphosphates (dNTP) incorporation in PacBio chips to deduce the presence of unusually modified nucleotides.

However, in both NGS (cluster sequencing) and NNGS (single-molecule sequencing) the nature of the signal may be only indirectly related to the chemical nature of the RNA modification, thus mis-identifications are not only possible, but actually rather frequent. In addition, if the analysis is performed at the whole-transcriptome scale (→10^6^–10^7^ nucleotides), even methods with an extremely good False Discovery Rate (FDR )<0.001 will still provide thousands of false positive hits. Thus, extreme care should be taken in the interpretation of large transcriptome-wide datasets claiming the presence of hundreds, or even thousands, of detected RNA modified nucleotides (as discussed in [1]).

The mapping of RNA modifications by NGS approaches is mainly based on: (1) altered base pairing during a reverse transcriptase (RT)-driven primer extension step, (2) altered chemical reactivity of the base due to a specific reagent (3) associated cleavage of the ribose-phosphate chain, and (4) a differential recognition of modified RNA nucleotides by specific antibodies (Ab) or proteins. In some instances, an enzymatic treatment or in vivo metabolic labeling can be used to exacerbate the chemical reactivity of a given modified nucleotide. Altered base-pairing is typically exploited for RNA modifications bearing extra chemical groups at the Watson–Crick (WC) edge of the base (so-called direct RT-signature) or for ‘RT-silent’ modifications after chemical derivatization affecting their WC edge. Methods that are based on the specific cleavage of the phosphodiester bond either rely on altered recognition by a specific enzyme or on the formation of an RNA abasic site, followed by a specific (and highly selective) ligation step. Antibody (protein enrichment) protocols exploit differential noncovalent or covalent binding to the modified site using UltraViolet light (UV) or chemical cross-linking steps.

Current approaches using NNGS (single-molecule sequencing) are mostly based on the use of direct nanopore RNA sequencing. Indeed, the profile of ion current registered for modified nucleotides passing through the nanopore is substantially altered when compared to the unmodified counterparts. While the experimental setup is generally rather straightforward and the RNA treatment is not different from classical RNA-Seq analysis, the extraction and analysis of raw nanopore sequencing data require complex and time-consuming bioinformatics treatment. Because of these limitations, NNGS methods are only at the emerging stage and cannot be considered to be an alternative to replace established NGS protocols. This will certainly evolve in the nearest future, and experience that accumulated in now-routine NGS analysis will be extremely helpful in the development of NNGS single-molecule analysis.

The analysis of RNA modifications by NGS is still a very recent topic (the first publications in the field are from 2012); however, a number of excellent review articles have already covered previous achievements [2,3,4,5,6,7]. In this comprehensive review, we will focus on the most recent and emerging approaches that have appeared during the last 2–3 years, and thye are not yet fully included in the relevant review literature.

## 2. Analysis of RNA Modifications by NGS

Different principles are currently employed for the detection of RNA modifications in the epitranscriptome using NGS and NNGS. We classify them in: (1) an analysis of RNA signatures that are visible in sequencing profiles (natural/enhanced or chemically induced), (2) the treatment-induced cleavage of the RNA phosphodiester chain followed by a selective ligation of sequencing adapters, and 3) affinity-based enrichment protocols exploiting the specificity of polyclonal or monoclonal antibodies and specific enzymes installing modifications in RNA. In many instances, the developed protocols use a combination of different principles (such as Ab-driven enrichment, followed by specific chemical treatment).

### 2.1. Naturally Existing RT Signatures of Modified Nucleotides

**Figure 1:**
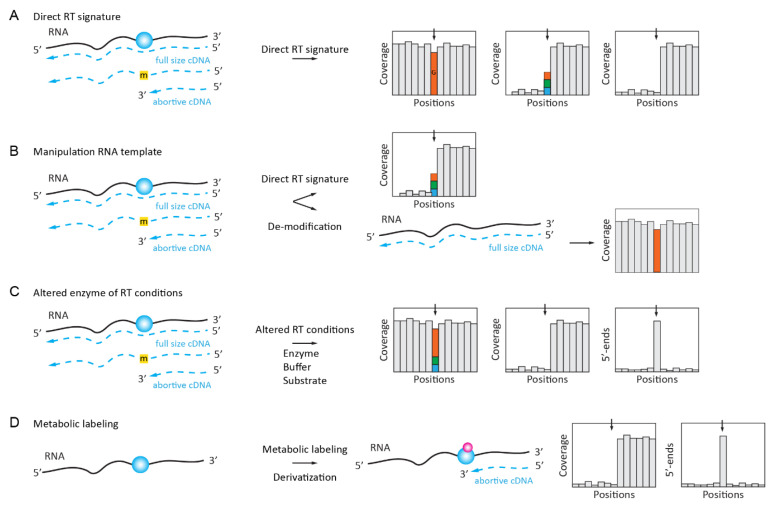
Direct detection of RNA modifications by natural or induced reverse-transcription (RT) signatures. (**A**): natural abortive/mutational RT signatures formed in cDNA. The signature may be seen as a pure mutation (different nucleotides are shown by color), a combination of abortive cDNA synthesis with mis-incorporation, or as a simple abortive cDNA synthesis. (**B**): reverse transcription signature is altered after the de-modification of RNA template. (**C**): RT signature is induced by specific RT conditions (buffer, substrate concentration), by altered deoxynucleotide triphosphates (dNTP), or by a mutation in the RT active site. (**D**): RT signature induced by metabolically labeled RNA-modified nucleotide (applied to RNA methylations with SAM analog).

Natural RT signatures consist of the altered reading of the modified nucleotide during primer extension by an RNA-dependent RNA polymerase (reverse transcriptase, RT). Depending on the nature of the nucleotide, such a signature may represent a “mutation” as compared to the expected reference sequence or constitute a more complex profile composed of mis-incorporations at different proportions in addition to the abortive RT products that end at the modified nucleotide. Such RT signatures can be manipulated by the pre-treatment of the RNA template (e.g., by the de-modification/removal of modified residues), by the choice of a particular condition of the primer extension, or by the use of non-natural dNTP substrate(s) and mutants of the RT active site. The in vivo metabolic labeling described later in this review also allows for the incorporation of reactive chemical groups at positions of certain modifications (methyl groups) and, thus, also helps to alter the RT signature of the modified RNA template (Figure 1D).

#### 2.1.1. Inosine “Mutation” RT Signature

The adenosine-to-inosine (A-to-I) deamination editing events in RNAs are catalyzed by enzymes from the ADAR/ADAT family. Because inosine base pairs with C and not with U (T) anymore, such events show up as A to G substitutions when the sequences of the genomic DNA and the cDNA are compared (Figure 1A). Such “mutational” RT signatures are the most straightforward approach for the detection of inosine residues in RNA. However, since the A-to-I conversion may only be partial, these inosine-generated “mutated” sequences are sometimes difficult to distinguish from sequencing errors or real SNPs in genomic DNA. Despite this limitation and the relatively high false detection rate, such a direct detection of the inosine residues is frequently used as an experimental design for the global analysis of the A-to-I editome [8,9].

#### 2.1.2. Complex RT Signatures for m^1^A (m^3^U, m^3^C, m^2^_2_G, etc.)

Unfortunately, only inosine shows a clear “mutational” RT signature out of →150 known RNA-modified nucleotides; all of the others are either totally silent during RT primer extension or generate more complex profiles due to their altered base pairing at the WC base edge. Such RT signatures comprise both the mis-incorporation of different bases at the position of modification and a strong RT-stop at the modified position. The proportion of both events (mis-incorporation and stop) depends on the nature of the RNA modification as well as that of the surrounding sequence [10,11] (Figure 1A). In line with these considerations, the HAMR method (High-throughput Annotation of Modified Ribonucleotides) [12,13] was initially used to map some potential m^3^C, m^1^A, m^1^I, m^2^_2_G, and m^1^G sites in human tRNAs, of which a selection of predicted m^3^C sites was experimentally validated. Later, the application of a specific RT-signature was developed for m^1^A mapping in low-complexity RNA (such as rRNA and tRNAs) [10,11] and, more recently, the analysis of m^1^A-generated RT-signatures was extended to the analysis of the human transcriptome [14]. 

Even if a simple RT signature approach is now considered to be moderately reliable, the concept of “RNA-modification fingerprints” is still under further developments using ultra deep sequencing datasets allowing for the characterization of more complex events, such as deletions and truncations that are generated at the position of RNA modification [15]. tRNA molecules that contain a large proportion of RNA modifications affecting cDNA synthesis by RT seem to be good candidates for such analysis, as was demonstrated by a comprehensive analysis of *Escherichia coli* tRNA modifications using an optimized pipeline for library preparation and analysis [16].

### 2.2. Enzymatically Enhanced Natural Signatures

The use of the natural RT signatures of RNA-modified nucleotides is straightforward and it does not require additional treatment steps, which might introduce uncontrolled biases. However, the real application of such methods is relatively limited, since additional controls are required to guarantee that the observed signal indeed corresponds to RNA modification and it is not an artefact due to the RNA 2D structure, local sequence context, or the presence of co-expressed tRNA isoforms only differing at a few positions. Thus, more advanced approaches exploiting RT signatures now include additional treatment steps that ensure that the observed signal is indeed RNA modification-dependent (Figure 1B). Such modulation of the RT signature can be achieved by the approaches that are described below.

#### 2.2.1. Manipulation (De-Modification) of the RNA Template

The enzymatic removal of methyl groups from some RNA-modified nucleotides can be achieved by demethylase enzymes of the AlkB type. This was demonstrated for the m^1^A, m^3^C, and m^1^G RNA modifications, which are commonly found in tRNAs. The profiles obtained for native and de-methylated templates are compared and de-methylated residues are detected by the disappearance of the RT signature after enzymatic treatment (Figure 1B). The application of such an approach to tRNAs leads to AlkB-facilitated RNA methylation sequencing (ARM-Seq) [17] and demethylase tRNA sequencing (DM-tRNA-Seq) [18]. More recently, another AlkB variant (D135S/L118V) protein was shown to efficiently and selectively de-methylate m^2^_2_G in tRNAs, thus improving tRNA deep sequencing and allowing for a more specific detection [19]. While both ARM-Seq and DM-tRNA-Seq share a similar de-modification concept, the experimental approach used is slightly different. ARM-Seq uses WT AlkB, while the demethylation enzymes used in DM-tRNA-Seq are optimized to broaden its specificity (including to m^1^G); moreover, the ligation-based library preparation protocol that is used in ARM-Seq only captures mutational signatures. The use of a thermophilic reverse transcriptase (TGIRT) and a template switch in DM-tRNA-Seq allows for the analysis of both RT arrest and mis-incorporation and, thus, may be preferred for analysis. De-modification is an attractive option, but the spectrum of enzymatically de-modified RNA modifications is limited to only few known substrates of alpha-ketoglutarate-dependent dioxygenases from the AlkB group. The successful application of DM-tRNA-Seq was described for the analysis of human microbiome tRNAome. De-methylation was found to improve the representativity of tRNA species and improve reliability in the assignment of Watson–Crick face base modifications [20].

An alternative approach for the manipulation of the RNA template was proposed in the fat-mass and obesity-associated (FTO)-assisted m^6^A selective chemical labeling method (termed m6A-SEAL) protocol [21]. Here, RT-silent m^6^A residues are first enzymatically oxidized to hm^6^A, followed by the chemical conversion of these unstable intermediates to N6-dithiolsitolmethyladenosine (dm^6^A) by DDT-mediated thiol addition. The resulting SH-containing modified bases can be labeled by biotin, pulled down by streptavidin beads, and they have their RNA fragments converted to a library and sequenced. The analysis is conducted similarly to the classical RNA immunoprecipitation (RIP) protocol.

The particular sensitivity of RNA exonucleases to the presence of modified RNA nucleotides was exploited in m6Am-Exo-Seq [22]. In this protocol, mRNA is uniformly fragmented and treated with a 5’ exonuclease to eliminate uncapped fragments, which results in a pool of capped mRNA 5’-end fragments enriched for m^6^Am and depleted for internal m^6^A. This selective enrichment is followed by de-capping and anti-m^6^A-immunoprecipitation (m^6^A-RIP). The resulting RNA fragments are analyzed by high-throughput sequencing.

#### 2.2.2. Manipulation of the Conditions for Enzymatic Reaction or the Nature of dNTP Substrate

The parameters of an enzymatic reaction (temperature, pH, concentration of monovalent/divalent metal ions, and dNTP substrates) can affect the properties of an RT enzyme upon the reading of some “RT-silent” RNA modifications [23,24] (Figure 1C). For example, it was shown that ribose 2’-O-methylations can be detected as stops (or strong pauses) when the avian myeloblastosis virus (AMV) RT enzyme was used at highly reduced dNTP concentrations (1–5 µM final) during primer extension. This method was extensively used in the past for the mapping of 2’-O-Me groups in various rRNAs [25] and it was recently coupled to NGS for the mapping of 2’-O-Me residues [26], termed the 2OMe-seq protocol.

The modulation of divalent metal ions [Mg^2+^] can also affect the properties of RTs. This was explored for the detection of m^1^A, m^2^_2_G, m^1^G, and m^3^C by replacing Mg^2+^ with Mn^2+^ in the reaction buffer of four different RTs. The arrest rate and mis-incorporation profiles are both strongly modulated under these altered conditions, with an increase in nucleotide skipping (deletions). Every RT polymerase shows an individual sensitivity to increased [Mn^2+^] and it depends on the nature of the RNA modification tested, allowing for a more reliable detection [27].

Finally, the detection of RT-silent RNA modifications can be achieved by the use of synthetic base-modified dNTP. Selenium substituted thymidine (4Se-dTTP) was found to induce RT stops at m^6^A due to the restricted base pairing and, thus, was used to generate a specific m^6^A signature in RNA [28].

#### 2.2.3. Manipulation of the RT Enzyme Properties

Native DNA polymerases with RT activity (from *Thermus thermophilus* in the original study) show a relative selectivity during the incorporation of nucleotides opposite m^6^A in the RNA template [29]. This observation was used for developing both low-throughput (single-base elongation- and ligation-based qPCR amplification method, termed SELECT protocol) [30] and high-throughput (Locus-specific Extension of Annealed DNA probes targeting m6A and sequencing, LEAD-m6A-seq) approaches [31]. In both of the methods, cDNA extension that is mediated by the Bst DNA polymerase allows for the distinction of unmodified and m^6^A-modified residues at selected loci.

The properties of RT enzyme can also be modulated by point mutations introduced at the enzyme active site (Figure 1C). This was explored with an engineered version of a thermostable KlenTaq DNA polymerase, which has a significant intrinsic RT activity. Generated mutant KlenTaq DNA polymerase displayed sensitivity to 2’-O-Me RNA residues, even at normal dNTP concentrations, and it can be used for the RTL-P-like [32] detection of modifications [33]. Another KlenTaq variant displayed sensitivity to m^6^A and exhibits increased mis-incorporation at respective sites of m^6^A in the template [34]. These engineered enzymes have not yet been coupled to the NGS detection of these modifications.

A similar approach was also applied for the selection of HIV-1 RT variants showing a particular sensitivity towards N1-methyladenosine (m^1^A). The selected mutant carrying six amino acid replacements shows both a robust RT primer extension activity and a well-defined RT signature at the modified position, suitable for the mapping and quantitative assessment of m^1^A modification [35].

#### 2.2.4. Manipulation of RNA Template by in Vivo Metabolic Labeling

A proof of concept for metabolic RNA labelling while using the S-adenosyl-L-methionine (SAM) analog was first demonstrated by the enzyme-catalyzed transfer of alkynyl moiety onto RNA [36]. However, the efficiency of transfer was quite low and further improvements were required for in vivo application. 

Such an application was developed for m^6^A mapping in cellular RNAs by the combination of SAM analog bearing the biorthogonal propargyl group and the “click-chemistry”-driven biotin labeling of the modified RNA molecules. For in vivo labeling, the SAM analog was replaced by propargyl-L-selenohomocysteine (Figure 1D). This enables the detection of METTL3 target sites by NGS as RT stops [37].

Another variant of SAM-analog metabolic labeling was also proposed to detect m^6^A at the transcriptome-wide level with single-nucleotide resolution and called ‘m6A-label-seq’. The living cells were fed with Se-allyl-L-selenohomocysteine, which substitutes the methyl group on the enzyme cofactor SAM with the allyl group. Allyl-modified a^6^A undergoes cyclization upon iodine treatment and Cyc-A is detected by NGS as RT mis-incorporation sites [38].

A number of recent reviews and surveys describe the use of biorthogonal metabolic labeling for the detection of RNA and RNA modifications [5,39,40,41].

### 2.3. Chemically Induced RT Signatures or RT Stops

#### 2.3.1. N1-Alkylation of Inosine (I)

**Figure 2:**
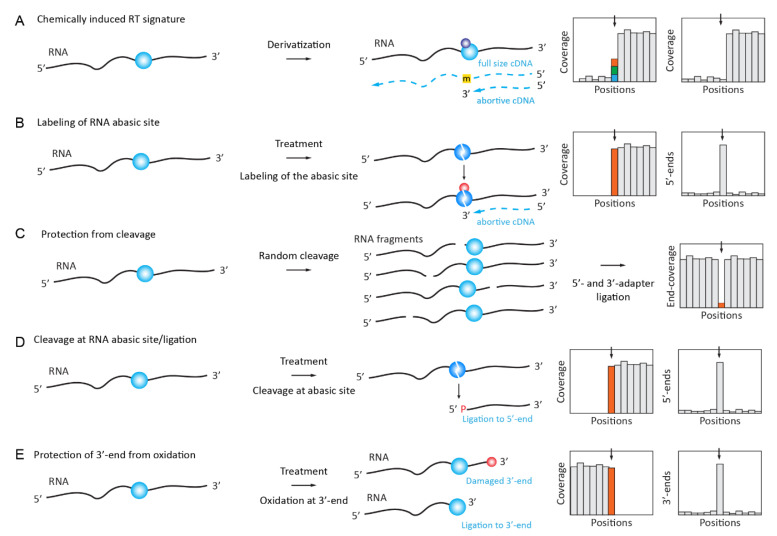
Detection of RNA modifications using chemically induced RT signatures or specific cleavage of the RNA ribose-phosphate backbone. (**A**): RNA treatment by a specific chemical reagent induces RT signature at the modified RNA residue in the form of a mis-incorporation profile (different nucleotides are shown by color) or abortive cDNA synthesis products. (**B**): chemical treatment selectively removes RNA-modified base by the cleavage of the N-glycosidic bond, releasing an RNA abasic site which can be labeled using an aldehyde-specific probe. (**C**): RNA modification induces the protection of the RNA backbone against cleavage (negative detection in RiboMethSeq and HydraPsiSeq protocols), thus excluding fragments starting and ending at the modified nucleotide. The signal is seen as a characteristic “gap” in 5’/3’-end coverage. (**D**): chemical treatment induces the cleavage of the RNA backbone and the release of a 5’-phosphate, which is a competent in 5’-adapter ligation. Panel (**E**): RNA modification (here, 3’-terminal Nm residues released by random RNA fragmentation) protects RNA from NaIO_4_ oxidation, thus maintaining the efficient ligation of the 3’ adapter.

The detection of inosine can be achieved on the basis of its A→G RT signature in the NGS data, as described above. However, multiple false-positive hits due to sequencing errors and SNPs contaminate such datasets. Treatment with acrylonitrile was shown to chemically modify inosines by alkylation, leading to RT arrest at inosine sites (Figure 2A) and ultimately allowing for the identification of inosines in a transcriptome-wide search with increased confidence (ICE-Seq [42]).

Another inosine labeling approach uses bulky acrylamidofluorescein for selective N1-inosine derivatization. Such labeling does not require subsequent reactions for affinity capture with specific antibodies and it can be used for the comprehensive transcriptome-wide analysis of A-to-I editing [43].

#### 2.3.2. S4-Alkylation of 4-Thiouridine (s^4^U)

Thio-substitued uridine at position 4 is a natural modified nucleotide that is found at position 8 in bacterial tRNAs, but it is rather known as an artificially introduced random RNA modification widely used in Photoactivatable Ribonucleoside-Enhanced Crosslinking and Immunoprecipitation (PAR-CLIP) experiments [44]. It was well known that the reverse transcription profile of s^4^U displays a certain percentage of mis-incorporation events, resulting in a U→C transition in the cDNA. The alkylation of s^4^U with iodoacetamide (ICH_2_-CONH_2_) that is used in SLAM-seq [45] alters the hydrogen binding pattern of s^4^U, which causes s^4^U to be retrotranscribed as a C, increasing the apparent transition rate to near-quantitative. While this method was mostly applied to trace the incorporation of s^4^U nucleoside into a nascent RNA in metabolic labeling experiments, it is certainly suited to trace posttranscriptional s^4^U modifications in bacterial tRNAs.

In another sequencing approach (named TUC-Seq), s^4^U was first introduced by metabolic labeling followed by conversion to cytidine residues by osmium (OsO_4_) treatment, thus abolishing the RT signature and allowing for a direct assessment of s^4^U-labeled RNA [46].

#### 2.3.3. Deamination and Oxidation of 5-methylcytosine (m^5^C)

The conversion of C-to-U residues in DNA and RNA by bisulfite is probably the most used chemical reaction in nucleic acid chemistry. Bisulfite RNA sequencing, which was adapted from 5mC detection in DNA, was described for m^5^C mapping and quantification by Schaefer and Lyko [47]. The method is generally considered to be relatively robust for abundant RNAs, such as tRNA and rRNA [48,49,50,51], but the reliability of the data obtained on less abundant RNAs, including mRNAs and lncRNAs [52,53,54,55], is still being debated [56,57,58].

Optimized and presumably more reliable bisulfite conversion protocols have been proposed with improved C-to-U conversion rates in the presence of formamide [58] and with a preferential amplification of only C→U converted RNA using a random ACT-containing DNA primer for the RT step [57].

As a further extension of RNA bisulfite deamination chemistry, approaches for the detection of f^5^C in RNA were suggested based on DNA-related protocols [59,60]. This nucleotide is not distinguishable from C by bisulfite treatment, but it can be reduced to hm^5^C by NaBH_4_, and hm^5^C in RNA is detected by its resistance to deamination, similarly to m^5^C. Alternatively, f^5^C is first protected by *O*-ethylhydroxylamine (fCAB-Seq for RNA), and this adduct becomes resistant to subsequent deamination [61].

A bisulfite-free single-base-resolution mapping protocol for m^5^C/hm^5^C in RNA was also suggested. It uses the selective oxidation of hm^5^C to trihydroxylated-thymine (thT) that is mediated by peroxotungstate. The thT residue base pairs with A during the RT step and, thus, becomes T in sequencing. If combined with the TET-mediated oxidation of m^5^C to hm^5^C, this method is also suitable for base-resolution m^5^C mapping [62].

Of note, in DNA 5hmC can be oxidized to f^5^C by potassium oxoruthenates (K_2_RuO_4_), followed by an aldol-type addition-elimination-cyclization sequence [63], and the ensuing alteration of base-pairing properties was exploited for sequencing. However, this technique still awaits potential adaptation to hm^5^C detection in RNA.

Recently, the global deamination of all RNA bases by nitrous acid treatment was used for m^6^A mapping and quantification. The protocol, termed NOSeq [64], exploits the resistance of m^6^A to chemical deamination under conditions where all other RNA nucleotides are efficiently converted. The protocol allows for targeted sequencing for the confirmation of m^6^A’s presence and the relative quantification of the modified residue.

#### 2.3.4. Derivatization of Pseudouridine (Ψ) by Soluble Carbodiimide

Classical approaches for RT-silent pseudouridine (Ψ) detection are based on the use of soluble carbodiimide (*N*-Cyclohexyl-*N*’-(2-morpholinoethyl)carbodiimide metho-*p*-toluenesulfonate, usually abbreviated as CMCT) [65] and they were developed in the 1990s for low-throughput Ψ mapping. Several groups successfully coupled this protocol to NGS almost simultaneously and independently [66,67,68,69,70,71,72]. A promising variant of the method used a “clickable” CMCT derivative for the subsequent enrichment of derivatized, pseudouridine-containing RNA [73,74]. Open questions include the moderate overlap of pseudouridine sites from different studies [75], potentially a consequence of variable sequencing depth (discussed in [76]). Carbodiimide-modified pseudouridine (CMC-Ψ) is generally detected as a strong RT stop, but sequence-dependent mutational signatures can also be detected and used for a more reliable detection of the modification [77].

Alternative chemistry for Ψ detection in RNA was proposed in RBS-Seq, the protocol that uses RNA bisulfite deamination under optimized conditions. In the presence of Mg^2+^ ions, the Ψ-monobisulfite adduct undergoes heat-induced ribose ring opening and Mg^2+^-assisted reorientation, causing base skipping during cDNA synthesis. These 1–2 base deletions in the read sequence can be detected and used for precise Ψ mapping by NGS [58].

#### 2.3.5. Dimroth Rearrangement of 1-Methyladenosine (m^1^A) to m^6^A under Alkaline Conditions

Under alkaline conditions, m^1^A, which causes mis-incorporation and abortive cDNA synthesis [10], undergoes Dimroth rearrangement to RT-silent m^6^A. This reaction was essentially used for validation by observing the disappearance of m^1^A RT-signatures in RNA incubated under alkaline conditions at high temperatures [14,78].

#### 2.3.6. Sodium Borohydride Reduction of 4-Acetylcytidine (ac^4^C)

For the 4-acetylcytidine (ac^4^C), which is present in tRNAs and rRNAs, a chemical reduction with sodium borohydride (NaBH_4_) leading to the saturation of the 5,6 double-bond in acetylated cytidines was employed. Reduced ac^4^C, in turn, produced ~20–30% of mis-incorporation signals in cDNA, as determined by Sanger sequencing [79,80]. This protocol was further improved by using sodium cyanoborohydride (NaCNBH_3_) under acidic conditions. This new mapping approach did not confirm previously reported mRNA ac^4^C data, but revealed a temperature-dependent modulation of rRNA/ncRNA and mRNA acetylation from hyperthermophilic archaea [81].

#### 2.3.7. Sodium Borohydride Reduction of 7-Methylguanosine (m^7^G)

A selective chemical reaction for m^7^G reduction by NaBH_4_, followed by aniline cleavage, was popularized in the 1970s [23,82,83,84]. However, this protocol was only recently applied to m^7^G detection using NGS. 

Samples were treated with NaBH_4_ to generate a visible RT signature from this otherwise RT-silent RNA modification, but subsequent aniline cleavage was omitted to generate RNA abasic sites with mutational signatures during RT primer extension. The method, called m^7^G Mutational Profiling sequencing (m7G-MaP-seq), allows for the high-throughput detection of m^7^G modifications at a single-nucleotide resolution [85].

The same NaBH_4_ reduction of m^7^G was used for its profiling in human miRNAs, but the resulting RNA abasic site was used for Schiff base formation with Biotin-ARP (Aldehyde Reactive Probe) reagent containing the reactive NH_2_ group for the selective enrichment of the modified sites (Borohydride Reduction sequencing, BoRed-seq) [86] (Figure 2B). The presence of m^7^G in miRNA is still under debate [87,88]. 

### 2.4. Chemically Induced Cleavage of the Ribose-Phosphate Backbone and Selective Ligation

#### 2.4.1. Detection and Quantification of Nm Residues by RiboMethSeq

Several variants of the so-called RiboMethSeq approach, which were first published by the Nielsen lab and further independently developed by two other groups [89,90,91,92], are based on a relative protection of the phosphodiester bond in RNA when the 5’-neighboring ribose is 2’-O-methylated (Figure 2C). These protocols are now widely used for the analysis of rRNA and tRNA 2’-O-methylations in different biological systems, including various pathologies [93,94,95,96,97,98,99].

#### 2.4.2. Detection of 7-methylguanosine (m^7^G) by TRAC-Seq and AlkAnilineSeq

The reduction of m^7^G with NaBH_4_, followed by aniline-induced cleavage (see above), led to the scission of the tRNA chain at the modified nucleotide and the position of the cleavage can be followed as RT-stop in RNA-Seq data in a combination termed TRAC-seq [100,101]. Demethylation by AlkB was used to remove major tRNA modifications, and the method was also coupled to m7G-RIP to enrich the modified targets.

Another recently established technique for m^7^G detection, named AlkAnilineSeq [102], relies on an alternative approach for the selective fragment enrichment of abasic sites, which were created by alkaline hydrolysis at positions containing m^7^G (as well as by m^3^C, D, and ho^5^C). Aniline cleavage results in downstream RNA fragments with 5’-phosphate, and a selective 5’-primer ligation ultimately led to a positive selection of fragments and, thus, to an exquisite sensitivity (Figure 2D). 

#### 2.4.3. Mapping and Quantification of Pseudouridine (Ψ) by HydraPsiSeq

Like the CMCT-based approaches that are described above, the recent HydraPsiSeq protocol exploits the resistance of pseudouridine residues to hydrazine cleavage while unmodified Us are readily cleaved under these conditions [83,103]. Conceptually, this method is a mid-point between AlkAniline-Seq (for the ligation of 5’-phosphates in RNA) and RiboMethSeq, since it uses a similar concept of ”negative” detection by protection against cleavage. The obtained signal directly reflects the pseudouridine level and it can be used for precise quantification. Normalization to natural unmodified RNA and/or to synthetic in vitro transcripts allows for the absolute measurement of modification levels. HydraPsiSeq requires minute amounts of RNA (as low as 10–50 ng), which makes it compatible with the high-throughput profiling of diverse biological and clinical samples.

#### 2.4.4. Profiling of m^3^C Using Hydrazine Cleavage

The same chemical reagent, hydrazine, was also proposed for the specific mapping of m^3^C residues in RNAs [104]. The technique of HAC-Seq uses preferential hydrazine-driven cleavage at m^3^C sites, followed by the decomposition of RNA abasic site by aniline, as in the AlkAniline-Seq and HydraPsiSeq protocols. Because other nucleotides (mostly U, see above) are also sensitive to hydrazine, a comparison between native and AlkB-treated (to remove m^3^C residues) samples is mandatory.

#### 2.4.5. Detection of Nm RNA Residues by Their Resistance to IO_4_ Oxidation (RibOxi-Seq/Nm-Seq)

Two conceptually similar methods, Nm-Seq [105] and RibOxi-Seq [106], exploit the stability of 2’-O-Me ribose against periodate (NaIO_4_) oxidation. RNA is first randomly fragmented either chemically (Zn^2+^) through cleavage at 95 °C (Nm-Seq) or enzymatically by benzonase (RibOxi-Seq), (de-phosphorylated if necessary) and subjected to 3’-terminal ribose oxidations by NaIO_4_. 3’-terminal Nm residues resist cleavage and remain competent for the ligation of 3’-adapter, while unmodified riboses are converted to a non-ligatable dialdehyde structure (Figure 2E). Because only a few (if any) 3’-Nm are directly exposed after a random cleavage, the removal of at least one (RibOxi-Seq) or multiple (Nm-Seq) terminal nucleotide(s) by successive NaIO_4_ oxidation/aniline treatment/de-phosphorylation is strictly required. Better enrichment is achieved when multiple cycles (up to eight cycles used in Nm-Seq protocol) of 3’-terminal nucleotide removal are performed. In comparison, Nm-Seq provides a better sensitivity in Nm detection, but it requires substantial amounts of input RNA, due to the inevitable loss occurring during NaIO_4_-oxidation/aniline treatment/de-phosphorylation steps.

#### 2.4.6. Selective Protection of m^6^A Methylated Motifs against MazF Cleavage (MAZTER-Seq)

The systematic quantitative profiling of m^6^A at a single-nucleotide resolution was achieved by use of MazF endonuclease, which is sensitive to the m^6^A methylation status of ACA motifs, overlapping with the classical DRACH (where D = A, G or U; R = A or G; H = A, C or U) motif for m^6^A modification by METTL3 [107]. RNA is totally fragmented by MazF, fragments are end-repaired, and the 3’-adapter is ligated. After a RT step, the second adapter is ligated to the cDNA 3’-end and the fragments are sequenced. Because of m^6^A-related protection, some ACA sites are absent from the library and this proportion can be quantified. 

### 2.5. Antibody-Based Enrichment Methods (MeRIP-Seq, i/miCLIP)

The use of specific antibodies for the detection of RNA modifications was proposed and successfully implemented in the late 1970s [108,109,110], and numerous polyclonal and monoclonal antibodies were used for analysis of DNA- and RNA-modified nucleotides (as reviewed in [111]). This development is still ongoing and highly specific antibodies can be obtained in this manner (see [112]). However, the majority of antibodies against modified nucleotides/nucleosides have a poor affinity and specificity [113] and, therefore, enrichment factors for modified RNA are only very modest [114]. Taking these considerations into account, it is not surprising that multiple artifacts in RNA modification mapping result from antibody cross-reactivity and uncertain specificity (as discussed in [115,116,117]). Despite these limitations, RNA modification-specific antibodies are widely used in the RNA Immunoprecipitation (RIP) and cross-linking and immunoprecipitation (CLIP) protocols that are applied in RNA modification mapping.

#### 2.5.1. RNA ImmunoPrecipitation (RIP) for m^6^A, m^1^A, hm^5^C, ac^4^C

**Figure 3:**
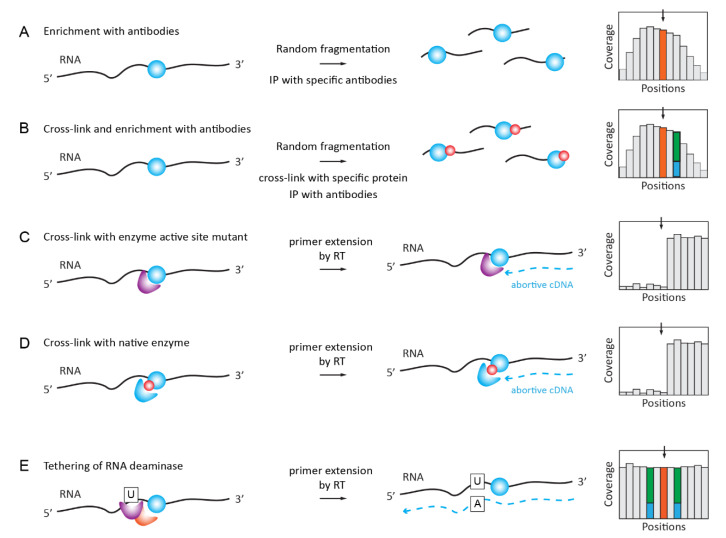
Detection of RNA modifications by immunoaffinity and other affinity-related methods. (**A**): A classical Me-RIP approach first applied to m^6^A and further extended also to non-methylated modified nucleotides. RNA is fragmented and the modification-containing fraction is enriched by immunoprecipitation. The signal of Me-RIP is somehow asymmetric, and a modified nucleotide may be displaced from the peak summit. Panel (**B**): immunoprecipitation-based individual-nucleotide resolution UV crosslinking and immunoprecipitation/Photoactivatable Ribonucleoside-Enhanced Crosslinking and Immunoprecipitation (iCLIP)/PAR-CLIP approaches using the covalent cross-linking of the specific antibody to RNA in the proximity of RNA modification. RT signature (shown in blue/green and resulting from RNA/protein cross-link may be visible in proximity to the modified nucleotide. Panel (**C)**: A covalent cross-linking with the modified residue when the enzyme active site mutant is used. Described for m^5^C and m^5^U methylating enzymes, which use a two-step reaction mechanism. Panel (**D**): cross-linking with the native enzyme may be achieved by the incorporation of non-natural nucleotides in RNA in vitro or in vivo (metabolic labeling). Panel (**E**): Reader protein for RNA modification is fused with the RNA deaminase domain (APOBEC) and, thus, tethered to the modified RNA residue (shown in orange), inducing partial C→U deamination in close proximity to RNA modification. These partial deamination signatures are visible in the sequencing profiles (shown in green/blue).

MeRIP-Seq is the most straightforward application of specific antibodies for RNA modification analysis. RNA is fragmented into pieces (100–150 nt in length), immunoprecipitated with RNA modification-specific antibody, and the enriched fragments are converted to libraries and sequenced (Figure 3A). A comparison with the profile that was obtained in the input fraction is mandatory and only broad enrichment peaks can be called, precluding a single-nucleotide resolution. The consensus sequence and the exact modification site inside the fragment are generally deduced by the conservation of the significantly enriched motifs. 

The applications of MeRIP-Seq for RNA modification detection are rather numerous, starting with cornerstone publications on m^6^A detection [118,119]. A similar approach was also applied for m^1^A mapping [78], as reviewed in [120]. More recently, MeRIP-Seq was also used for the mapping of hm^5^C [121], ac^4^C [122], and m^7^G [123].

#### 2.5.2. iCLIP/PAR-CLIP with Antibodies and Specific Proteins/Enzymes

Further improvements in MeRIP-Seq specificity and resolution were brought about by the inclusion of a UV-crosslinking step in addition to Ab-driven immunoprecipitation (Figure 3B). Specificity is improved by the use of more stringent wash conditions that are allowed by the covalent link between the RNA ligand and the Ab, and a single-nucleotide resolution is obtained due to the C→U mutational signature present in close proximity to the modification site. Successful applications of the iCLIP/miCLIP approaches include the mapping of m^6^A and m^6^Am [124,125], as reviewed in [126]. For specific m^6^Am detection by miCLIP, differential maps were generated using PCIF1 KO [127]. PAR-CLIP methods are based on the use of a photoactivatable nucleotide (generally s^4^U) and cross-linking by mild UV-light irradiation at 365 nm [128].

#### 2.5.3. Covalent Cross-Linking with RNA Target Mediated by RNA Modification Enzyme or Specific Reader Protein

The idea to use an RNA modification enzyme (or its catalytic mutant) for covalent attachment to the target sequence in RNA is based on a two-step mechanism of m^5^C-MTases, including two distinct steps: the methylation of the target base and the recycling of the covalent intermediate to release the enzyme from the covalent complex with RNA [129,130,131]. 

A high-throughput implementation was developed for human NSUN2 catalytic mutant (C271A) and it was called individual-nucleotide-resolution crosslinking and immunoprecipitation (iCLIP) protocol (Figure 3C). This allowed for the identification of numerous NSUN2 targets in coding and non-coding RNAs [132]. Recently, the same protocol was updated for mapping the RNA substrates of the *E. coli* radical SAM m^8^A-MTase RlmN by miCLIP-MaPseq [133]; for a review of catalytic crosslinking-based methods, see [134].

An alternative cross-linking protocol, called Aza-IP (AzaIP-Seq), uses 5-azacytidine-mediated RNA immunoprecipitation to form a covalent bond between an RNA substrate and native m^5^C-MTase in vivo [135]. Crosslinked targets are subsequently identified by high-throughput sequencing [136]. The use of thermostable group II intron reverse transcriptase (TGIRT) allows for the direct detection of the crosslinked nucleotides by mismatches [137]. 

The formation of covalent adducts with RNA substrate was also known for m^5^U_54_-MTase RUMT [138], and the incorporation of the 5-fluorouracil into the RNA substrate allows to trap covalent reaction intermediates [139]. Thus, the method Fluorouracil-Induced-Catalytic-Crosslinking-Sequencing (FICC-Seq) was used for a genome-wide single-nucleotide resolution mapping of human TRMT2A modification sites [140] (Figure 3D). 

Finally, the fusion of a m^6^A-reader YTH domain with a cytidine deaminase APOBEC1 allows for the specific binding of YHT to m^6^A-modified RNA and the site-specific APOBEC1-catalyzed deamination of adjacent C residues (Figure 3D). This Deamination Adjacent to RNA modification Targets (DART-seq) protocol has identified thousands of m^6^A sites in cells and it can detect m^6^A accumulation over time [141]. 

### 2.6. Data Analysis and Interpretation

An essential step in the application of all high-throughput procedures is the error-free base calling of the candidate modified sites and the application of appropriate statistical tests to evaluate the probability of false-positive/false-negative identifications [1,142]. Only recently have these aspects come to be seriously considered when many highly optimistic RNA modification datasets were released. There are a number of publications that provide such analyses and discuss the reasons for the poor accuracy and specificity of high-throughput applications; they also provide strategies that minimize false positives and other pitfalls that are associated with mapping and measuring epitranscriptomic modifications [142,143]. Another analysis points out a surprising number of experimental and bioinformatics artifacts, which can ultimately lead to substantially inflated estimates of the abundance of diverse modifications [144]. Such discrepancies being observed in the analysis of even identical experimental raw data led to an extensive discussion of the real number of m^1^A sites in the human transcriptome, showing that mis-annotations, mis-mapping, SNPs, and sequencing errors may greatly contribute to the over- or under-estimation of the real number of modifications [145,146].

Identical considerations of the accuracy of bioinformatics pipelines should be also applied to the analysis of RNA A→I editing [147] and m^5^C mapping in RNA by bisulfite sequencing [56,148]. 

The generalization of common and/or web-based bioinformatics pipelines that are adapted to RNA modification analysis will certainly improve the reliability and reduce the erroneous mis-identification of RNA-modified nucleotides [149,150,151,152].

## 3. Analysis of RNA Modifications by NNGS (Single-Molecule Sequencing)

The use of single-molecule sequencing approaches (NNGS or third-generation deep sequencing) is an attractive alternative to classical cluster sequencing protocols. Indeed, cluster sequencing involves an amplification step, providing only an average picture of modifications in a population of RNA molecules. Single-molecule analysis should be performed to obtain information regarding the exact combinations of modifications in a given RNA chain (individual modification pattern) [153].

The proof of principle for the analysis of RNA modifications (namely, m^6^A) by single-molecule sequencing was established seven years ago by using the PacBio single-molecule, real-time (SMRT) technology. HIV-1 and AMV RT were loaded to a zero-mode waveguide (ZMW) chip and the extension of DNA primer on the RNA template was monitored [154]. Even if the precision of the RNA sequencing remains limited, the analysis of the RT kinetics can be used to identify the RNA base modifications. This work was not pursued further, most probably because HIV-1 RT containing ZMW chips for PacBio machines is not commercially available. 

More recent examples of single-molecule RNA sequencing for the detection of RNA modifications concern direct RNA sequencing by nanopores (Oxford Nanopores). Using direct RNA sequencing, it was demonstrated that m^6^A RNA modifications can be detected with a high accuracy in the form of systematic errors and decreased base-calling qualities [155]. With appropriate training datasets containing m^6^A-modified and -unmodified synthetic sequences, the prediction of m^6^A RNA modifications can be achieved with ~90% accuracy. The analysis of ion current profiles for the direct MinION nanopore sequencing of full-length 16S rRNA revealed conserved and aminoglycoside antibiotic resistance-related 7-methylguanosines (m^7^G) as well as pseudouridine modifications [156]. 

It is clear that the major challenge in the field of direct RNA sequencing and RNA modification mapping by nanopores consists of the use of appropriate data analysis software and algorithms. Analysis can be either conducted by standard base calling and the identification of “sequencing signatures” or by the extremely laborious, but direct, analysis of ion current traces. The first solution is implemented in the software MINES (m^6^A Identification using Nanopore Sequencing), which assigns m^6^A methylation status to more than 13,000 previously unannotated DRACH (D = A/G/U, R = A/G, H = A/C/U) sites in endogenous HEK293T transcripts and identifies more than 40,000 sites with isoform-level resolution in a human mammary epithelial cell line [157].

The direct analysis of nanopore ion current profiles is extremely computationally heavy, but it certainly provides more valuable information. The bioinformatic tool, called Epitranscriptional Landscape Inferring from Glitches of ONT signals (ELIGOS), was trained on various types of synthetic modified RNA and applied to rRNA and mRNA sequencing. ELIGOS is able to accurately predict known classes of RNA methylation sites (AUC > 0.93) in rRNAs from *E. coli*, yeast, and human cells [158]. Model-based base calling from ionic current signal levels is certainly required for reliable analysis [159]. Another intermediate solution, a workflow for the analysis of direct RNA sequencing reads, termed MasterOfPores, converts raw current intensities into multiple types of processed data, including FASTQ and BAM, providing metrics of the quality of the run, quality filtering, demultiplexing, base calling, and mapping. In a second step, the pipeline performs downstream analyses of the mapped reads, including the prediction of RNA modifications and the estimation of polyA tail lengths [160]. 

In the context of the COVID-19 pandemic, the direct RNA nanopore sequencing of full-length coronavirus genomic RNA allowed for us to predict multiple sites of m^5^C modification in SARS-Cov-2 [161]. However, the existence of these modifications in SARS-Cov-2 is still controversial, since another study utilizing nanopore sequencing with more rigorous controls did not confirm their presence [162]. 

In conclusion, direct RNA modification analysis by nanopore sequencing is rapidly developing and improving in reliability, but it has still not reached maturity for routine application in RNA epitranscriptomics. Thus, classical approaches are still widely used in the routine analysis of RNA modifications, and the use of nanopores is only envisaged as an alternative validation technique.

## Data Availability

Not applicable.
